# *Candida* Antigen Titer Elevation and Mortality in Burn Patients

**DOI:** 10.29252/wjps.8.1.18.

**Published:** 2019-01

**Authors:** Sebastian Jachec, Walter Perbix, Perbix Fuchs, Rolf Lefering, Christian Weinand

**Affiliations:** 1 Department of Plastic, Reconstructive and Aesthetic Surgery, Hand Surgery, Burns, University Hospital Cologne, Merheim, Germany;; 2 Institute for Research in Operative Medicine (IFOM), University Hospital Cologne, Merheim, Germany;; 3 Department of Plastic and Aesthetic Surgery, Hand Surgery, Burns Helios Hospital Gifhorn, University of Magdeburg, Germany

**Keywords:** *Candida*, Burn, Mortality, CAG titer

## Abstract

**BACKGROUND:**

Mortality in burn patients has several contributing factors as sex, age, degree of burns, or inhalation injuries. Usefulness of *Candida* antigen (CAG) titer is still being under debate to predict mortality. This study assessed correlation between CAG titer and mortality in burn patients.

**METHODS:**

From 1988 to 2011, 877 burn intensive care patients were evaluated for age, sex, total burn surface area (TBSA), multi organ failure (MOF), burn depth, escharotomy, fasciotomy, antibiotic use, co-morbidities, CAG titer and intubation.

**RESULTS:**

From 870 admitted patients, 190 patients were not enrolled. Increasing age was correlated with a higher mortality. The abbreviated burn severity index (ABSI) score of the deceased was 4 points and the TBSA was 20% higher than the survivors. The correlation for age, intubation, TBSA, inhalation injury, MOF, CAG titer, antibiotic use and escharotomy was significant. An increasing mortality was noted with antibiotic use and a CAG titer of 1:8 and higher. CAG titer of 1:8 and higher had a sensitivity of 51.1% and specificity of 86.3% for mortality. Multivariate analysis confirmed high influence of older age, MOF, comorbidities, antibiotic use and CAG titer of 1:8 and higher on mortality. There was a significant correlation for sex, younger age and CAG titer.

**CONCLUSION:**

CAG titers of 1:8 and higher might warrant beginning of antimycotic treatment in elderly patients with high TBSA to avoid increase in mortality.

## INTRODUCTION

Mortality of burn injuries increases with higher total burned surface area and occurring infections.^1^ Burn patients are susceptible to infections, because of the loss of the natural skin barrier and immuno-compromise. Burn size and defect are proportionate with odds of suspecting an infection.^2^ As bacterial infections can be detected with relative ease, suspicion of existing fungemia is found to be high in burn patients because of the existing immune suppression.^3^ Before the use of PCR or real-time PCR, CAG titer was widely used for detection of candidemia and showed its impact on finding the diagnosis. Nowadays, different methods have been developed. Current non-culture methods rely on a polymerase chain reaction (PCR) assay for candidemia.^4^

Real-time PCR methods open up new perspectives for the early diagnosis of low candidemia, as an adjunct to blood culture.^5^ Real-time PCR has considerable advantages over conventional PCR in terms of sensitivity, handling, reduced contamination problems and has so far outperformed the CAG titer in diagnosing candidemia. Furthermore, it can be used to quantify the amount of template DNA and has been used to measure fungal loads.^6^^,^^7^ However, in none of the scores nowadays used fungal blood stream infection is being considered in calculating morbidity and mortality for the burn patient population. This is where measuring CAG titer might become a useful tool to predict increased mortality. Especially in the burn patients population who are susceptible to fungal blood stream infections, a swift and fast interference could be life-saving. Therefore, additional evaluation is mandatory. In our study, we evaluated the influence of the presence of CAG titer of 1:8 and higher on the mortality of a burn population of 870 patients, compared with other factors mentioned in literature.

## MATERIALS AND METHODS

From January 1988 to December 2011, in 877 burn patients with thermic, electrical or chemical burns, admitted to our burn intensive care unit (BICU), CAG-titer was measured. Management was not changed significantly for patients during this time period. The inclusion criteria were sustained burn injury and burn intensive care needed, age over 14 years, no previous treatment outside our institution, ABSI data, and at least two CAG titers measured. Exclusion criteria were previous nystatin prophylaxis.

For evaluation several data were available, especially data on concomitant diseases of every patient, process of the burn injury and other sustained injuries, calculated abbreviated burn severity index (ABSI), all microbiological data of every patient, all CAG titers of all patients and data on catecholamine use, respiratory therapy and FiO_2_, use of blood and blood products, and antibiotic and antimycotic therapy. Mortality of patients was the mortality of all the patients during and after burn intensive care treatment during the hospital stay. Patients who were discharged to other hospitals for further therapy were not included in the study. 

In our hospital, the CAG titer was measured twice per week, using the latex agglutination test.^7^ When a patient showed increasing signs of infection in the laboratory (i.e. increasing white blood cell count, increasing c-reactive protein, increasing need of circulation supporting medication and increasing temperature) with MOF the titer was used for evaluation of the necessity of an antimycotic therapy.

A 1:2 CAG titer was ignored. If sepsis could not be explained otherwise and except for patients with pressure sores who usually showed CAG titers of 1:4 to 1:8, a titer of 1:8 often resulted in the use of antimycotic therapy. In patients with titers of 1:16 and higher, antimycotic therapy was always started. Antimycotics were chosen according to the antimycotic resistogram and during microbiology rounds, provided by the Department of Microbiology. In addition to antimycotic therapy, dressings were changed more often and topical antifungals were applied such as amphotericin B, or betaisodona and all catheters were exchanged. 

The SPSS program (version 16.0, Chicago, IL, USA) was used for statistical analysis. For statistical evaluation univariate (age, sex, MOF, TBSA, burn depth, escharotomy, fasciotomy, antibiotic use, co-morbidities, and intubation), Chi-square and Fischer Exact test, multivariate model analysis, positive and negative predictive value, finite model analysis and the Nagelkerke and Cox and Snell R-square tests were used. The tested end point was non-survival. Differences were considered significant for a *p*<0,05.

## RESULTS

870 patients were included into evaluation. When grouping into age groups, the first age group contended the highest number of patients (427 patients), the last groups, the lowest number of patients (37 patients). Increasing age showed higher mortality. Organ failure was grouped into no organ failure (489 patients), one organ failure (127 patients) and two or more organ failures (254 patients).

First patients were separated into non-survivors and survivors (Table 1), while 190 patients did not survive during the hospital stay (21.8%). The mean age of non-surviving patients was 50 years, and the survivors 40 years. The mean ABSI score was 4 points higher in the non-survivor group than in the survivor group, total TBSA was also 20% higher in the non-survivor group than in the survivors. Non-survivors had a higher percentage of 2nd and 3rd degree burns than survivors (Table 1). 

**Table 1:** Burn patient population in this study

**Variable**	**Non-survival**			**Survival**		
	**n**	**Mean**	**SD**	**n**	**Mean**	**SD**
Age	190	50.58 years	20.83	680	40.06 years	17.95
ABSI	190	9.56	2.31	680	6.35	2.15
TBSA	190	43.90%	23.83	680	23.05%	16.76
2a degree	167	11.98%	12.17	638	13.56%	11.35
2b degree	171	16.54%	11.8	502	7.81%	9.27
3rd degree	170	19.92%	13.6	372	7.60%	9.89

When second univariate analysis was performed, the chosen endpoint was non-survival, and Chi-square and Fischer exact test were performed, variables tested were age groups in steps of 20 years, sex, intubation, TBSA in steps of 10% increased burned surface area, MOF, inhalation injury, CAG titer in doubling steps up to 1:16, co-morbidites, antibiotic use, escharotomy and fasciotomy. Co-morbidities and sex had the least influence on the outcome, CAG titer had a significance of 0.0001, comparable to the other tested variables (Table 2).

**Table 2:** Univariate analysis of factors influencing mortality, Exitus=mortality

**Variable **	**Non-survival** **n (%) **	**Chi-Square**	**Fischer Exact test**
	190 (21.8)		
Age [years] (Total)		0.0001	0.0001
0-19	12 (13.3)		
20-39	51 (15.1)		
40-59	65 (23.1)		
60-79	46 (36.8)		
80 and higher	16 (43.2)		
Sex (total)		0.094	0.1
Male	128 (20.4)		
Female	62 (25.6)		
Intubation (total)		0.0001	0.0001
No	7 (2.8)		
Yes	183 (29.6)		
TBSA (total)		0.0001	0.0001
0-9%	11 (6.9)		
10-19%	19 (8.4)		
20-29%	33 (20.0)		
30-39%	25 (24.0)		
40-49%	27 (31.8)		
50-59%	28 (51.9)		
60-69%	17 (59.0)		
70-79%	11 (50.0)		
80% and higher	19 (90.5)		
MOF (total)		0.0001	0.0001
None	18 (3.7)		
1 organ	15 (11.8)		
2 or more organs	157 (61.8)		
Inhalation injury (total)		0.0001	0.0001
No	62 (14.6)		
Yes	128 (28.7)		
CAG titer (total)		0.0001	0.0001
1:1	27 (7.6)		
1:2	29 (16.3)		
1:4	37 (25.0)		
1:8	55 (45.5)		
1:16	42 (60.9)		
Co-morbidities (total)		0.003	0.004
None	61 (16.9)		
1 ormore	129 (25.3)		
Antibiotic		0.0001	0.0001
No	30 (12.6)		
Yes	160 (25.4)		
Escharotomy		0.0001	0.0001
No	97 (15.5)		
Yes	93 (38.0)		
Fasciotomy		0.001	0.001
No	164 (20.4)		
Yes	26 (39.4)		

When the influence of antibiotic use on CAG titer was evaluated, use of antibiotic treatment led to a higher number of patients with CAG titer and there was no increase in CAG titer height with the use of antibiotic (Table 3). The next step was testing the sensitivity and specificity of CAG titer lower than 1:8 and 1:8 or higher for death and the positive and negative predictive value. The sensitivity was 51.1%, specificity was 86.3%. The positive predictive value for a CAG titer of 1:8 and higher for impending non-survival in our burn patients was 51.1%, while the negative predictive value for CAG titer being below 1:8 for survival was 86.3% (Table 4). 

**Table 3:** Crosstable on influence of antibiotic treatment and CAG titer

**Variable**	**n (%)**	**CAG titer**					**Total**
		**1:1**	**1:2**	**1:4**	**1:8**	**1:16**	
Antibiotic use	No n (%)	153 (64)	36 (15.1)	23 (9.6)	15 (6.3)	12 (5.0)	239 (100)
	Yes n (%)	201 (31.9)	142 (22.5)	125 (16.8)	106 (16.8)	57 (9.0)	631 (100)
Total	n (%)	354 (40.7)	178 (20.5)	148 (17.0)	121 (13.9)	69 (7.9)	870 (100)

**Table 4:** Sensitivity (51.1%), specifity (86.3%), positive predictive value (51.1%) and negative predictive value (86.3%) of CAG titer 1:8 or higher on mortality

	**Variable**	**CAG-titer**		
Non-survival		Below 1:8	1:8 or higher	Total
	No n (% of non-survivors) (% of CAG-titer 1:8)	587 86.3% 86.3%	93 13.7% 48.9%	680 100.0% 78.2%
	Yes n (% of non-survivors) (% of CAG-titer 1:8)	93 48.9% 13.7%	97 51.1% 51,1%	190 100.0% 21.8%
Total	n (% of non-survivors) (% of CAG-titer 1:8)	680 78.2% 100.0%	190 21.8% 100.0%	870 100.0% 100.0%

Multivariate analysis of the variables showed a significance of *p*<0.022 for CAG titer of 1:8 and higher. Intubation, no MOF or one organ failure, inhalation injury, escharotomy and faschiotomy were not found to be significant. The Nagelkerke test for variance was r²=0.611, showing a positive correlation for the tested variables (Table 5). In the calculated final element model those variables were included that were significant in the previous multivariate analysis. 

**Table 5:** Multivariate analysis, endpoint of the evaluation is death, r² (Nagelkerke)=0.611 for variance

**Variable**	**P value**
Age [years]	
0–39	0.025
40–59	0.0001
60–79	0.0001
80 and higher	0.0001
Sex	0.012
Intubation	0.298
TBSA	0.0001
MOF 1	0.056
MOF 2 and more	0.0001
Inhalation injury	0.172
CAG titer 1:8 andhigher	0.022
Co-morbidites	0.0001
Antibiotic	0.006
Escharotomy	0.539
Fasciotomy	0.154

Inhalation injury was also evaluated, because this was a variable known to influence death as outcome in burn population. Age group of 0-19 and 20-49 were combined for evaluation. CAG titer of 1:8 and higher had a significance of 0.021 with a coefficient of 1.7, as high as the tested variables sex and age group of 0-49 years. The highest weight in this model had MOF of two or more organs with a coefficient of 3.1 and a significance of 0.0001. The inhalation injury did not have any influence on the outcome variable of non-survival in our model (Table 6). 

**Table 6:** Finale element model with coefficient B for tested variables, standard variation of error (SE), Odds ratio (OR), 95% confidence interval for OR and significance of variables (Sig). The CAG titer has comparable weight like the sex or younger age for mortality

**Variable**	**B**	**SE**	**OR**	**95% confidence Interval for OR**	**P value**
**Age **					
40-59	0.67	0.279	1.955	1.133-3.375	0.016
60-79	2.069	0.358	7.915	3.921-15.978	0.0001
80 and higher	2.212	0.558	9.130	3,060-27.239	0.0001
Sex	0,635	0.278	1,886	1.094-3.253	0.022
TBSA	0.042	0.006	1.043	1.031-1.056	0.0001
MOF 1	0.765	0.408	2.149	0.966-4.781	0.061
MOF 2 and more	3.122	0.318	22.698	12.180-42.297	0.0001
Inhalation injury	0.367	0.251	1.443	0.882-2.359	0.144
CAG titer 1:8 and higher	0.570	0.247	1.769	1.091-2.869	0.021

## DISCUSSION

In this study, we evaluated the usefulness and the weight of the CAG titer on prediction of mortality in burn patients. Burn patients are cited as being among the high risk groups for invasive fungal infections.^8^ Burn wounds provide an ideal port of entry for invasive infection, while also inducing substantial immune dysfunction.^9^ The risk increases with burn size ^10^ and the extent of burn body surface has been correlated with immune suppression and gastrointestinal mucosal atrophy, favoring Candida translocation.^3^

In our study, we found an increase of mortality with age and burn size, but also an increase of mortality with increased CAG titer during the use of antibiotics. In a previous study, we have shown a positive correlation between the use of antibiotics, candidemia and mortality.^11^ The importance of bacteremia and prior antibacterial therapy as risk factors for fungal invasive infections was also conﬁrmed by the study of Costa-de-Olivera, as half of the patients had positive bacterial blood cultures before the ﬁrst fungemia episode and 93% of them had received wide-spectrum antibacterial drugs.^12^ This might explain the high coefficient in our finale element model. 

Data available from the Burn Unit of the University Hospital of Coimbra regarding fungal infections reported from 856 patients admitted from January 2003 to November 2007, 69 (8%) developed fungal infections. A higher mean age, a greater percentage of total burned body surface area, and a longer hospital stay in the burn unit were registered in patients who developed fungal infections.^13^^,^^14^ These findings are concordant with the results in our study.

According to Cheng *et al.*,^15^ the most important risk factors for *C. albicans* candidemia are advanced age, invasive procedures associated with intensive care, and acute sepsis and, for non-albicans species, the most important risk factors were cancer chemotherapy in association with leucopenia and thrombocytopenia, the latter being a common ﬁnding among critical burn patients. 

However, these factors and others like the CAG titer, which have critical weight in the assessment of intensive burn care patients, are not taken into account in current scores used for critically ill or burn patient population such as the ABSI score, the Belgian score or the Flames score.^1^^,^^16^^,^^17^ These burn scores included several factors such as sex, age, TBSA, 3rd degree burns, inhalation injury, heart rate, arterial pH, mean arterial pressure, creatinine, hematocrit white blood cell count, Glasgow coma scale, rectal temperature and AaDO2. We therefore calculated, based on the majority of these burn score factors, the sensitivity and specificity of the CAG titer to predict mortality. 

According to a previous study,^4^ PCR detection of fungal DNA had a sensitivity of 72.1%, a speciﬁcity of 91.2%, and a negative predictive value of 93.2%, when compared with blood culture. For patients with known predisposing factors for candidemia, who have negative blood cultures, a positive PCR result from blood may represent a reliable evidence of invasive candidosis, thus providing a rationale for the initiation of targeted antifungal therapy.^4^ In our study, we calculated for the CAG titer a sensitivity of 51.1%, a specificity of 83.6% and a negative predictive value of 86.3%. 

However, although considerable efforts are being developed along the last couple of years to validate non-culture methods and molecular assays as diagnostic tools for fungal blood stream infections, the lack of standardization and/or reduced speciﬁcity and sensitivity still precludes its routine diagnostic use.^6^ Additionally, in clinical settings, where diagnosis of candidemia leans heavily on the use of real-time PCR or PCR while these tests are not available, clinical importance of CAG titer should not be underestimated.^7^

The fast and reliable specification and susceptibility testing are important tests to be performed for all fungal isolates, including *Candida* obtained from sterile sites, from urine of burn patients in ICU, and from wounds. In fact, a progressive increase in colonization almost invariably predicts the development of invasive candidosis.^18^^,^^19^ An invasive candidosis might lead to a life threatening candidemia, especially in immune-compromised patients.^7^ Here, a CAG titer of 1:8 and higher might be a valuable predictor to an increased risk for mortality in burn patients. However, an increasing CAG titer by itself has also been reported a Dumping–phenomenon in a previous study.^19^ It might be therefore necessary to take all clinical variables of the patient into account. The CAG titer might be considered a useful adjunct in the assessment of mortality in burn patients.
